# A highly effective and inexpensive standardized treatment of multidrug-resistant tuberculosis: a multicenter prospective study in China

**DOI:** 10.1186/s12879-021-06553-2

**Published:** 2021-08-19

**Authors:** Wenwen Sun, Zheyuan Wu, Ying Zhou, Fan Xia, Qin Tang, Jie Wang, Jinghui Yang, Fangyou Yu, Hua Yang, Heping Xiao, Lin Fan

**Affiliations:** 1 Department of Tuberculosis, Shanghai Pulmonary Hospital, Shanghai Clinical Research Center for Tuberculosis, Tongji University School of Medicine, Shanghai, 200433 China; 2 Shanghai Municipal Center for Disease Control and Prevention, Shanghai, China; 3 Shanghai Xuhui District Central Hospital, Shanghai, China; 4 Department of Pulmonary Disease, PLA 905 Hospital, Shanghai, China; 5 Shanghai Key Lab of Tuberculosis, Shanghai Pulmonary Hospital, Shanghai Clinical Research Center for Tuberculosis, Tongji University School of Medicine, Shanghai, China; 6 Department of Clinical Laboratory, Shanghai Pulmonary Hospital, Tongji University School of Medicine, Shanghai, China

**Keywords:** MDR-TB, Treatment regimen, MIC, Treatment outcome, Adverse effects

## Abstract

**Background:**

To verify the efficacy and safety of an inexpensive standardized regimen for multidrug-resistant tuberculosis (MDR-TB) with low resistance to isoniazid (INH), a multicenter prospective study was conducted in eastern China.

**Methods:**

Patients diagnosed as MDR-TB with low concentration INH resistance and rifampicin resistance, second-line/injectable agents sensitive were prospectively enrolled, given the regimen of Amikacin (Ak)–Fluoroquinolones (FQs)–Cycloserine (Cs)–Protionamide (Pto)–PasiniaZid (Pa)–Pyrazinamide (Z) for 6 months followed by 12 months of FQs–Cs–Pto–Pa–Z, and then followed up for treatment outcomes and adverse events (AEs).

**Results:**

A total of 114 patients were enrolled into the study. The overall favorable treatment rate was 79.8% (91/114). Among 91 cases with favorable treatment, 75.4% (86/114) were cured and 4.4% (5/114) were completed treatment. Regarding to unfavorable outcomes, among 23 cases, 8.8% (10/114) had failures, 8.8% (10/114) losing follow up, 0.9% (1/114) had treatment terminated due to intolerance to drugs and 1.8% (2/114) died. Treatment favorable rate was significantly higher in newly treated MDR-TB (91.7%, 33/36) than that in retreated MDR-TB (74.4%, 58/78, *p* 0.03). The investigators recorded 42 AEs occurrences in 30 of 114 patients (26.3%). Clinicians rated most AEs as mild or moderate (95.24%, 40/42).

**Conclusions:**

The regimen was proved to be effective, safe and inexpensive. It is suitable for specific drug resistant population, especially for newly-treated patients, which could be expected to be developed into a short-course regimen.

*Clinical trials registration* China Clinical Trial Registry ChiCTR-OPC-16009380

## Background

Multidrug-resistant tuberculosis (MDR-TB) is a major global health problem with treatment success rate less than 60% [1]. The 2019 WHO consolidated guidelines recommended three kinds of therapeutic options (all-oral long/short or injection included) for countries and programs treating MDR-TB [1]. However, varied status in different areas or countries resulted in varied outcomes, depending on different factors such as financial support, management, protocol, drug quality, newly included drugs, patient compliance and tolerance. Therefore, how to achieve the optimal treatment outcome for MDR-TB and ensure its high safety has become the most important issue for clinicians. The all-oral regimen containing bedaquiline (BDQ) instead of injectable agents recommended in 2019 guideline [2] may be effective but difficult to be implemented in resource-poor areas due to its high price. It was pointed out that the explicit recommendations for the use of Linazolamide (LZD) and BDQ might bring some disadvantages to the global TB control program [3]. The ideal treatment for MDR-TB should be effective, with fewer adverse events (AEs) and affordable, especially in resource-poor areas with high TB burden [4].

According to the WHO 2019 report, the treatment coverage rate of MDR-TB in China was only 13.6% [1]. Delayed drug sensitivity test (DST), unaffordable treatment costs due to long courses or expensive drugs and AEs were the main reasons [5]. The WHO guideline in 2016 recommended a shorter regimen for patients without second-line drug resistance which had been proved to be effective in multiple countries, called “Bangladesh regimen” [6, 7]. On the other hand, MDR-TB had multiple factors which can influence treatment outcome including previous treatment history or resistance patterns [8]. Therefore, we designed a treatment regimen similar to the regimen recommended by the 2016 WHO guidelines according to the national conditions based on DST results. In the present study, we used minimum inhibitory concentration (MIC) drug sensitivity test (DST) to evaluate the drug resistance for certain TB drugs that could make cost effective treatment regimens.

This prospective clinical trial was conducted in three TB specialist hospitals from the eastern China to evaluate and verify the safety and efficacy of an inexpensive standardized regimen on MDR-TB patients with at least isoniazid (INH) low resistance and Rifampin resistance through a multicenter prospective study. We hope to explore a relatively inexpensive and effective regimen for MDR-TB patients with specific drug resistance pattern. The results of the study might provide a treatment model for MDR-TB in source limited areas with high TB burden, individualized stratified treatment of MDR-TB based on MIC DST.

## Methods

### Study design

The study is an open-label, prospective cohort study. All patients received treatment for 18 months. They were followed up for at least 1 year after end of the course. All patients were recruited from three regional tuberculosis hospitals and Shanghai Center for Disease Control and Prevention (CDC). These institutions referred patients to Shanghai Pulmonary Hospital, a national TB clinical treatment center with nearly 300 beds receiving referral of patients with MDR-TB from eastern China, for further treatment. In addition, the hospital is the only specialized hospital receiving MDR-TB in Shanghai and undertakes the region formulation and management collaborated with Shanghai CDC.

Based on previous studies [7, 9, 10], estimated the size of the study, at the based 94 estimation that the favorable treatment rate were 70%. Approximately at least 80 participants are needed to make up the sample size, with a unilateral type I error of 0.05 and 20% missed follow-up.

### Study patients

From January 2017 to June 2018, we enrolled patients between 18 and 65 years old diagnosed as MDR-TB referred from 3 TB specialist hospitals and from general hospitals who reported to Shanghai CDC.

Inclusion criteria were patients satisfied with all following conditions: Patients with MDR-TB confirmed by MIC DST at least resistant to INH at low concentration and rifampicin (R) resistance within 2 months prior to screening; and MDR-TB patients with no injectable agents or FQs resistance; and MDR-TB patients previously only received first-line anti-TB treatment (ATT) or had no previous history of ATT or previous history of second-line ATT less than 1 month.

Excluded criteria were as follows: Extensively drug-resistant tuberculosis (XDR-TB) patients (resistance to both FQs and second-line injection agents) or pre-XDR (resistant to either FQs or second-line injection agents); MDR-TB patients with any serious systemic diseases or immune diseases; with extrapulmonary tuberculosis; co-infected with HIV and other virus; taking immunosuppressive agents; history of FQs use for more than 1 month in the recent 6 months; pregnant, adolescent and infant.

We defined the newly treated MDR-TB as having never received ATT or having been on treatment for less than 1 month [9]; Re-treated cases were defined as registered MDR-TB patients who had previously taken first-line anti-TB drugs for more than 1 month; or if there is sufficient documentary evidence of having been treated with first-line anti-TB drugs for 1 month or more in the past [11].

### Inclusion and intervention

Patients meeting the inclusion criteria were given the regimen consisted of Amikacin (Ak), Fluoroquinolones (FQs), Cycloserine (Cs), Protionamide (Pto), Pasiniazine (Pa) and Pyrazinamide (Z) for 6 months intensive phase and the continuation phase consisted of FQs, Cs, Pto, Pa and Z for 12 months continuation phase.

All patients were screened within 1 week of receiving the first dose and administered under directly observed therapy (DOT) throughout the treatment course. During the course of treatment, patients were visited by the same specialist every 2 weeks until the end of the course. Visits were made every 3 months after completion of treatment until 12 months after completion of treatment.

### Minimum inhibitory concentration (MIC) of the drugs

Sputum/bronchoalveolar lavage samples were collected for MTB culture at the following times: at baseline; at the end of every month until the end of the treatment.

All strains were tested by MIC DST, the drugs were isoniazid (INH) and Rifampicin (R) as well as Pa, Ak, Mfx (Moxifloxacin) and Lfx (Levofloxacin). M. tuberculosis H37Rv (ATCC 27294) was used as a control strain. Laboratory steps were performed by trained persons in biosafety cabinets according to the relevant guidelines. Samples were pretreated with some steps and were inoculated in Middlebrook 7INH9 broth supplemented with 10% ADC and 0.05% Tween-80 (Sigma) and incubated at 37 °C for 3–4 weeks. The following steps were in accordance with the state guidelines. The MIC value was defined as the lowest drug concentration that inhibited growth of bacteria. The MIC value of a strain to certain drug higher than (≥) the cut-off concentration is resistant to this drug. The cut-off concentration were 1 µg/mL for Pa, 5 µg/mL for Ak, 1 µg/mL for Rifampicin (R), 0.2 µg/mL for INH, 0.5 µg/mL for Moxifloxacin and 1 µg/mL for Levofloxacin.

### Treatment efficacy evaluation

Treatment outcomes for MDR-TB were evaluated according to WHO guidelines [12]. The treatment outcomes were divided into “cured”, “completed treatment”, “failure”, “default” and “death”. The “cured” was referred as patients completed treatment with consistently at least five negative culture results for the final 12 months of the treatment course and without evidence of treatment failure [13]; “Completed treatment” was determined by bacterial negative conversion at the end of the treatment with less than three negative cultures. The ‘‘failure’’ was referred as patients had sputum culture positive in the final 12 months of the treatment course or if any one of the final three cultures was positive or to be discontinued due to clinical or radiological adverse reactions or adverse events. ‘‘Death’’ was patients died from any reason during the course of ATT; ‘‘Default’’ was referred as patients whose TB treatment was interrupted for at least 2 consecutive months for any reason. Favorable treatment outcomes were defined as sum of “cured” and “treatment completed”, unfavorable outcomes included “failure”, “default” and “death” [14].

### Safety assessment

Adverse events (AEs) were defined and graded by monthly checkups including routine blood counts, biochemical tests and urine tests. AEs endpoints included all-cause mortality and incidence of adverse events that occurred or worsened during treatment, defined by the Division of Microbiology and Infectious Diseases [15]. The severity of AEs and the need to discontinue the relevant drugs were determined by the TB specialist.

### Statistical analysis

The statistical analysis was conducted with SPSS 18.0 (IBM Corp, Armonk, NY, USA). The baseline data were compared between newly treated and retreated groups. Classification variables were described as frequency and percentage, and compared using a Chi-square test or a Fischer’s exact test. Continuous variables are described as medians. When the data are normally distributed, the t-test was used to compare the mean values of continuous variables, otherwise, Mann–Whitney test used. Chi-square analysis was used to compare treatment outcome, sputum conversion rate and the incidence of AEs. Kaplan–Meier curves were used to calculate the cumulative percentage of sputum bacteria conversion within 1 year of treatment initiation, and the results were determined by Log-rank, Breslow, and Tarone tests. A difference was considered as significant if the *p* value was less than 0.05.

## Results

### Study population

During the study period, 130 MDR-TB received eligible evaluation, 16 patients were excluded due to 5 cases with pre-XDR-TB, 3 cases with extrapulmonary TB, 1 case with poor compliance, 3 cases with having severe complications and 4 cases not willing to participate (4 cases). A total of 114 patients were finally included into the study, including 72 males with median age at 35.7 years (range 19–64 years) and 42 females with median age at 32.2 years (range 19–61 years), 36 cases with newly treated MDR-TB and 78 cases with retreated MDR-TB. The baseline characteristics of the patients enrolled in this study are summarized in Table 1.

**Table 1:** Baseline clinical characteristics of patients enrolled

Characteristic	Newly treated (n = 36)	Retreated (n = 78)	p value
Age (mean ± SD)	32.1 ± 12.4	35.5 ± 11.5	0.13
Male n (%)	22 (61%)	50 (64.1%)	0.34
Body mass index (BMI), kg/m^2^ (range)	20.8 (12–30)	19.2 (14–29)	0.54
Cavities present on Chest CT			
No cavity	18 (50%)	36 (46.2%)	0.82
Unilateral	12 (33.3%)	35 (44.9%)	0.08
Bilateral	5 (13.9%)	7 (9.0%)	0.14
Lesion severity			
≥ 3 fields	22 (61.1%)	49 (62.8%)	0.55
< 3 fields	14 (38.9%)	29 (37.2%)	0.76
Complications			
DM	5 (13.9%)	10 (12.8%)	0.35
*AEs n (%)	6 (16.67)	24 (30.77)	**0.00

^*^AEs: Patients with adverse reactions

^**^The difference was statistically significant

### Treatment outcome of included patients

**Fig. 1:**
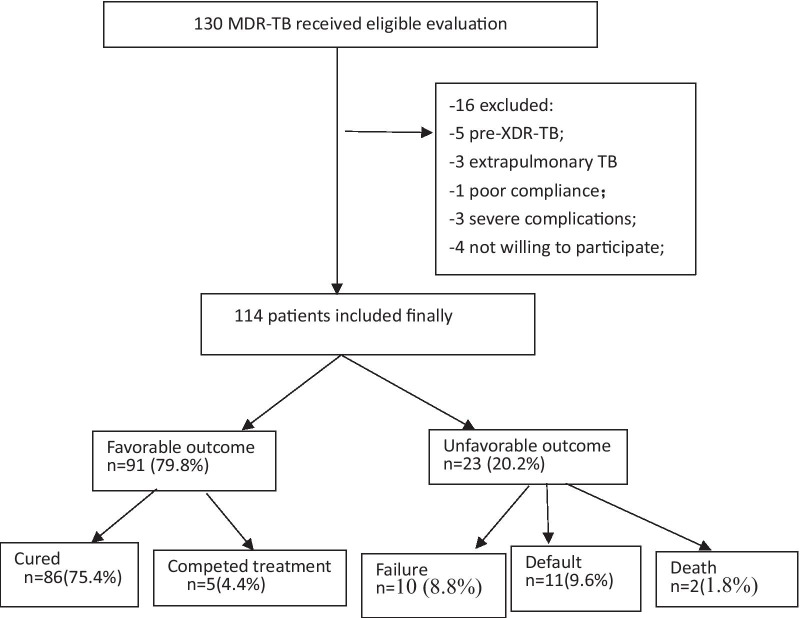
Flow diagram

The overall favorable treatment rate was 79.8% (91/114): 75.4% (86/114) cured, 4.4% (5/114) completed treatment. 20.2% (23/114) got unfavorable outcome: 8.8% (10/114) failures, 9.6% (11/114) default including 8.8% (10/114) losing follow up and 0.9% (1/114) withdrawing treatment due to intolerance to drugs; and 1.8% (2/114) died. The flow diagram of patients included was shown in Fig. 1

Treatment favorable rate was significantly higher in newly treated MDR-TB (91.7%, 33/36) than that in retreated MDR-TB (74.4%, 58/78), *p* value was 0.03.

**Fig. 2:**
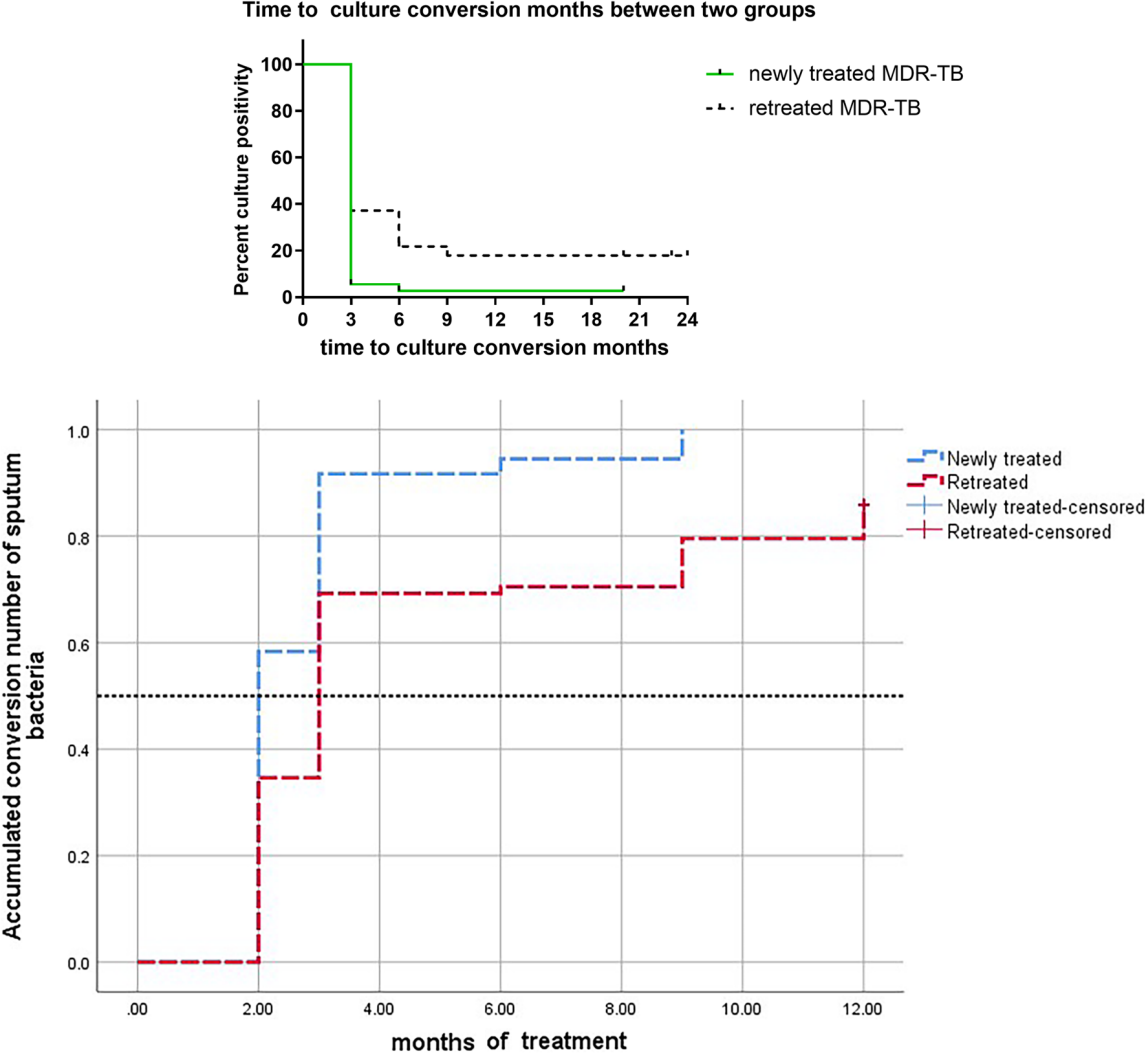
**a** The time to culture conversion was significantly shorter in newly treated MDR-TB than that in retreated MDR-TB. **b** Sputum culture negative conversion rates in newly treated group were significantly higher than that of retreated group; median time to sputum transformation was 2 months in the newly treated group and 3 months in the retreated group

Another key analysis assessed the difference in sputum culture conversion time between newly treated and retreated group. Table 2 shows the Kaplan–Meier curve of the cumulative proportion of culture-converted patients in different treatment groups. Sputum culture negative conversion rates at the end of the 3rd month and the 6th month were 91.7% (33/36) and 94.4% (34/36) in newly treated group which were significantly higher than that of 69.2% (54/78) and 70.5% (55/78) in retreated group, *p* value was 0.009 and 0.004, respectively (Fig. 2a, Tables 2, 3). And median time to sputum transformation was 2 months in the newly treated group and 3 months in the retreated group (Fig. 2b).

**Table 2:** The difference in sputum culture conversion rates of two groups during the first 12 months of the treatment

	Chi-square	df	Sig
Log rank (Mantel–Cox)	12.775	1	.000
Breslow (Generalized Wilcoxon)	9.368	1	.002
Tarone–Ware	10.802	1	.001

Test of equality of survival distributions for the different levels of statistic methods

The p values of the three test statistics were all < 0.05, there were significant differences in the sputum culture conversion rate between the two groups

**Table 3:** The differences of time to culture conversion between two groups

	Newly treated (n = 36)	Retreated (n = 78)	p value
Sputum negative conversion rate at month 2 (n, %)	15 (41.67)	18 (23.08)	0.00
Sputum negative conversion rate at month 3 (n, %)	33 (91.67)	54 (69.23)	0.01
Sputum negative conversion rate at month 6 (n, %)	34 (94.44)	55 (70.51)	0.00
Achieve the standard of stopping medicine at the end of 12 month	26 (72)	25 (32)	0.00

A total of 51 (44.73%, 51/114) patients achieved the standard of stopping medication [11] at the end of the 12th month: among which 26 (72%, 26/36) patients were newly treated, and 25 (32%, 25/78) were retreated, *p* value was 0.00.

All patients who completed the course of treatment were followed for 1 year and no recurrence was found.

### Safety and adverse events (AEs)

The investigators recorded 42 AEs occurrences in 30 of 114 patients (30/114, 26.3%). Clinicians rated most AEs as mild or moderate (n = 40 AEs, 40/42) and no one was caused by Pa (0/114, 0%). The incidence of AEs were significantly lower in newly treated patients (6/36, 16.67%) than those in retreated patients (24/78, 30.77%).

Among them, 22 AEs occurrences in 15 patients (13.16%, 15/114) experienced a change in treatment regimen. Among them, 12 AEs occurrence in 10 patients associated with Ak due to mild hearing loss (n = 5) or mild renal dysfunction (n = 7). 6 AEs occurrences in 3 patients associated with Z due to transaminase elevation (n = 3, alanine aminotransferase were elevated to 2 times higher than the upper limit of the normal range) and gout (n = 3). 4 AEs occurrences in 2 patients associated with Pto due to transaminase elevation (n = 2, alanine aminotransferase were elevated two times higher than the upper limit of the normal) and gastrointestinal reaction (n = 2). All AEs above were improved after discontinuation of the suspected drugs. 9 (60%, 9/15) of these patients returned to the discontinued drug after dosage adjustment and completed the course.

4 AEs in 2 patients (1.75%, 2/114) were reported to be severe enough to require permanent discontinuation of the suspected drug: 1 case (0.88%, 1/114) was associated with Ak by hearing loss (n = 1) and mild renal insufficiency (n = 1). Allergic rash (n = 1) and gastrointestinal reaction (n = 1) caused by Mfx in 1 case (0.88%, 1/114). Patient with allergic skin rash were unable to tolerate and abandoned the treatment.

**Fig. 3:**
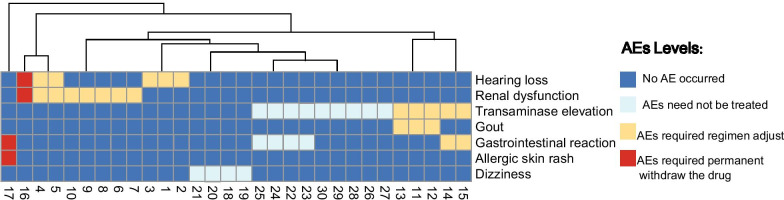
Heatmap of the AEs composition (Lines) of the 30 patients (Columns). 42 AEs were found in 30 patients, and some patients may have more than one AE. The X axis represents 30 patients, the Y axis represents the type of the AEs, and the different colors represent the degree of the AEs. There will be several clusters for each patient

As the rest 16 AEs occurrences in 13 patients (11.40%, 13/114) were improved after symptomatic treatment, and no treatment plan was changed: 4 AEs occurrences in 4 patients suspended Ak due to mild dizziness (n = 4); 12 AEs occurrences in 9 patients suspended Z and Pto due to mild transaminase elevation (n = 8, alanine aminotransferase elevated 2 times lower than the upper limit of the normal range.) and gastrointestinal reaction (n = 4), See in hot map of AE in Fig. 3.

Among the 30 patients with AE, treatment success was in 25 patients (83.3%) including 5 patients from newly treated group and 20 patients from retreated group; unfavorable outcome was in 5 patients (17.7%), including 2 cases withdrawing treatment due to AE of the drugs, 1 cases withdrawing the treatment without specific reason, 1 case getting failure and 1 case loss of follow-up.

## Discussion

In the present study, we made up a regimen for the treatment on MDR-TB mainly according to guidelines of WHO 2016 [16]. We included MDR-TB patients with no resistance to FQs or injectable agents based on MIC DST. The importance of DST for individualized MDR-TB treatment has been well established, with resistance to key drugs associated with poor treatment outcomes [17]. The correlation between the MIC and the treatment outcomes of MDR-TB has also been verified by several studies [18, 19]. In the present study, the results showed that the overall favorable treatment rate was 79.8%, especially as high as (91.7%, 33/36) in newly treated patients. The incidence of AEs were also low, especially in newly treated patients (6/36, 16.67%) and the majority of AEs (n = 40 AEs, 40/42) were mild-to-moderate. The results demonstrated the high efficacy and safety of this regimen against specific MDR-TB patients. At the same time, the clinical guidance value of the DST of anti-tuberculosis drugs screened by MIC had positive guiding value for clinical practice.

Previous study shown that for patients without HIV infection, sputum culture negative conversion time can be regarded as an essential signal for successful treatment of MDR TB patients [20]. In the present study, we recorded sputum culture results every 3 months and plotted a Kaplan–Meier curve for patients with sputum culture cumulative conversion over the course of 1 year of treatment. The results showed that the sputum negative conversion rate of newly treated patients was significantly higher than that of retreated patients. Therefore, this regimen may be more suitable for newly treated patients. In addition, clinicians should pay close attention to the retreated patients who may have a worse prognosis.

The Bangladesh short range regimen recommended by the 2016 guidelines is suggested for treating MDR patients without FQs and second-line injectable agents resistance, which consisted of an intensive period of 4 to 6 months with seven drugs (Ak–FQs–Pto–Clofazimine (Cfz)–Z-high dose INH-E(Ethambutol) followed by a five-month course of FQs–Cfz–Z–E [21]. But Cfz is expensive in China (about $ 400/month) and may be unacceptable to most Asian patients due to the skin pigmentation, especially young women. In the present study, under inclusion conditions similar to the short range regimen: we replaced the high dose INH with Pa and replaced Cfz with Cs. The results indicated high favorable treatment outcome rate and the entire treatment regimen costs at only around $300 per month, while the short range regimen costs $450 per month and the all-oral regimen recommended by the 2019 WHO guideline costs more than $2000 per month in China. For resource-limited areas, the cost of treatment is an important determinant of patient compliance. Costly drugs, even if effective, may cause patients to discontinue treatment, which not only lead to the spread of drug-resistant TB, but can also lead to more complex drug resistance in individuals. China is a high TB burden country with high financial burden of health care. Therefore, BDQ had not been widely used at present for its price. On the other hand, the WHO’s clear recommendations on the use of LZD and BDQ could prove a double-edged sword for global TB control programmes [3]. Intuitively, the advantages of including LZD and BDQ in standard protocols for all types of MDR-TB may be more conducive to programme implementation and less likely to require DST. The main drawback, however, may be concerns about patient’s safety and tolerability. Lzd has significant long-term side effects as an ultra-broad-spectrum antibiotic and about 30–40% of patients stop linezolid treatment because of AEs [22]. Therefore, in the present study, patients were selected with inclusion criteria, and only FQs was selected as the included drug among class A drugs.

The rate of INH acetylated is controlled by genetics. Once acetylated, INH is ineffective as an antibiotic against TB bacilli. Rapid acetylation of INH may lead to low serum concentrations of anti-TB drugs, increasing the risk of treatment failure. Most Asians are of the fast-metabolizing type [23]. It had been reported that in the INH-resistant organisms studied, about 50% of INH MIC belong to the category of low concentration resistance with MIC at 0.1–1.0 ug/mL [24, 25]. A study from China showed that among 109 INH-resistant isolates, only 11.9% and 19.3% showed resistance to PAS and Pa, respectively [26]. Pa is a chemical synthesis of isoniazid (INH) and paminosalicylic acid (PAS). PAS effectively delays and blocks the acetylation of INH in vivo. Pa maintains high, prolonged concentration of INH in the blood and reduces toxicity to the liver. It not only enhances the bactericidal action of the drug, but also delays the generation of bacterial resistance. In the present study, patients infected with low concentration INH resistant strains were included, and MIC values showed that all strains were sensitive to Pa. Another advantage of Pa is its low price ($22 per month). Its safety was reflected in the incidence of AEs (0%).

Cycloserine (Cs) in group B has good antibacterial activity and the price in China is much lower than Cfz ($250 vs $400 per month). Due to its low drug resistance rate and low cross-resistance with other anti-TB drugs, it is often used as a good alternative drug for MDR-TB [27, 28]. The present study also verified its safety and efficacy.

Second-line injections (SLIs) were once one of the core drug groups in treatment of MDR-TB [16]. However, existing studies have shown that SLIs has high AEs and often leads to withdrawal of the treatment [29]. A retrospective study of 25 countries [30], according to the results of injection therapy (Ak) were better than no injections, but in analysis of comprehensive treatment results, patients who received the injection did worse than those who did not receive the injection. The results provided evidence for 2019 guidelines on the recommendation regarding the use of injectable agents [1]. But the article also explained that Ak may be the most widely used injectable drug due to its price and tended to be used in patients with the worst resistance patterns, which may be one of the reasons for poor outcomes rather than the problem with the drug itself. However, the present study showed that 18 AEs occurrences in 15 (13.16%, 15/114) patients suspicious of Ak due to mild hearing loss or mild renal dysfunction. But only 1 (6.67%, 1/15) patient had Ak permanently disabled. The results suggest that the Chinese population could be moderately tolerant to SLIs and that the cheap drug is certainly effective for certain populations.

Our study still had some limitations, including the relatively small sample size and the short follow-up period. Existing study shown that 3% of patients experienced MDR-TB recurrence during an average follow-up period of 4.8 years [31]. Therefore, it may be necessary to enroll more patients and follow up longer time to validate our conclusions.

## Conclusions

The regimen in the present study had the following characteristics: highly effective with favorable treatment rate in newly treatment patients reached 91%; Pa instead of high-dose INH and CS instead of Cfz in the treatment of specific MDR-TB populations may be more suitable for Chinese; It proved that the AEs of SLIs are controllable in Chinese population and inexpensive; 72% (26/36) newly treated patients achieved the standard of withdraw medicine at the end of the 12th month. These characteristics suggested that the regimen could be widely used in China, even other resource-poor parts of Asia. Further research on the possibility of short-course treatment in resource-poor areas with high TB burden could be expected.

## Data Availability

All data regarding the included participants and laboratory data during the study are available from the corresponding author by email request. The clinical study was registered at The China Clinical Trial Registry (ChiCTR, www.chictr.org.cn) with the Registration number: ChiCTR-OPC-16009380.

## Abbreviations

INH: Isoniazid
Ak: Amikacin
FQs: Fluoroquinolones
Cs: Cycloserine
Pto: Protionamide
Pa: PasiniaZid
Z: Pyrazinamide
LZD: Linazolamide
BDQ: Bedaquiline
DST: Drug sensitivity test
MIC: Minimum inhibitory concentration
CDC: Disease Control and Prevention
ATT: Anti-TB treatment
XDR-TB: Extensively drug-resistant tuberculosis
DOT: Directly observed therapy
Mfx: Moxifloxacin
Lfx: Levofloxacin
R: Rifampicin
AEs: Adverse events
Cfz: Clofazimine
E: Ethambutol
PAS: Paminosalicylic acid
SLIs: Second-line injections
MDRTB: Multidrug resistant tuberculosis
